# Distribution of E- and N-cadherin in subgroups of non-functioning pituitary neuroendocrine tumours

**DOI:** 10.1007/s12020-022-03051-6

**Published:** 2022-06-08

**Authors:** Kristin Astrid B. Øystese, Olivera Casar-Borota, Jon Berg-Johnsen, Jens Petter Berg, Jens Bollerslev

**Affiliations:** 1 Department of Endocrinology, Morbid Obesity and Preventive Medicine, Oslo University Hospital, Oslo, Norway; 2 Institute of Clinical Medicine, Faculty of Medicine, University of Oslo, Oslo, Norway; 3 Department of Immunology, Genetics and Pathology, Uppsala University, Uppsala, Sweden; 4 Department of Clinical Pathology, Uppsala University Hospital, Uppsala, Sweden; 5 Department of Neurosurgery, Oslo University Hospital, Oslo, Norway

**Keywords:** Pituitary neuroendocrine tumours, Non-functioning pituitary adenomas, Corticotroph pituitary adenomas, Adherence junctions, Prognostic markers of pituitary tumours

## Abstract

**Purpose:**

Clinically non-functioning pituitary neuroendocrine tumours (NF-PitNETs) present a varying degree of aggressiveness, and reliable prognostic markers are lacking. We aimed to characterise the distribution of E- and N-cadherin in corticotroph, PIT1 and null-cell NF-PitNETs, and link it to the course of the tumours.

**Methods:**

The distribution of E- and N-cadherin was investigated by immunohistochemistry in a retrospective cohort of 30 tumours of the less common NF-PitNETs (corticotroph (*N* = 18), PIT1 (*N* = 8) and null-cell PitNETs (*N* = 4)). Immunoreactive scores (IRS) were compared to previously presented cohorts of gonadotroph NF-PitNETs (*N* = 105) and corticotroph functioning PitNETs (*N* = 17).

**Results:**

We found a low IRS for the extra-cellular domain of E-cadherin (median 0 (IQR 0–0, *N* = 135)), a medium to high IRS for the intra-cellular domain of E-cadherin (median 6 (IQR 4–9)) and a high IRS for N-cadherin (median 12 (IQR 10.5–12)) throughout the cohort of NF-PitNETs. The corticotroph NF-PitNETs presented a higher IRS for both the extra- and intra-cellular domain of E-cadherin (median 0 (IQR 0–1) and median 9 (IQR 6–12), respectively) than the gonadotroph NF-PitNETs (*p* < 0.001 for both comparisons). Presence of nuclear E-cadherin was associated with a weaker staining for the intra-cellular domain of E-cadherin (median 4 (IQR 0.5–6) and median 9 (IQR 9–12), for tumours with and without nuclear E-cadherin, respectively), and with a lower rate of re-intervention (*p* = 0.03).

**Conclusions:**

Considering our results and the benign course of NF-PitNETs, we suggest that a high N-cadherin and downregulation of membranous E-cadherin are not associated with a more aggressive tumour behaviour in these subgroups of NF-PitNETs.

## Introduction

Clinically, non-functioning pituitary neuroendocrine tumours (NF-PitNETs) are common intracranial neoplasms, comprising nearly half of all PitNETs [1, 2]. The tumours are epithelial in origin, and classified histopathologically by the immunolabeling for anterior pituitary hormones and transcription factors (SF1, TPIT and PIT1), the null-cell adenomas stain neither for pituitary hormones nor for transcription factors [3]. The tumours are characterised as non-functioning due to their lack of clinical symptoms based on the lack of hypersecretion of anterior pituitary hormones [4].

Some studies have shown that the different histopathologic subgroups of NF-PitNETs present diverse clinical courses [5–7], with some tumour characteristics mimicking their hormone-producing counterparts [5]. The corticotroph NF-PitNETs (staining for ACTH and/or TPIT) have been found to show more aggressive features than the gonadotroph NF-PitNETs (staining for FSH, LH and/or SF1) [8, 9]. However, these findings are not consistent [10].

Epithelial tissue is characterised by a polarised cell orientation, a fixed position and stable cell-to-cell contacts [11]. Loss of epithelial differentiation is involved in the development of more invasive and metastatic tumours in the pathogenesis of several epithelial neoplasms and linked to the process of epithelial to mesenchymal transition (EMT) [12–15]. Cadherins are cell adhesion molecules, ensuring proper tissue architecture and cell functioning. The classical cadherins are transmembrane proteins comprising an extra-cellular domain, a transmembrane domain and an intra-cellular domain. Both E- and N-cadherins belong to the classical cadherins type 1 and share a similar structure. E-cadherin is highly expressed in epithelial tissue, and a downregulation is associated with a more aggressive phenotype in several epithelial cancers [16–18]. N-cadherin is prevalent in non-epithelial tissue, and an upregulation has served as an indicator of EMT [19, 20]. EMT is a complex and potentially reversible process where the tissue changes some of the epithelial features in favour of a more mesenchymal phenotype. This epithelial to mesenchymal plasticity enables the cells to adopt mixed epithelial and mesenchymal features, and to change phenotype at different stages of EMT [21].

Previous studies have investigated epithelial markers in PitNETs, pointing to an association between downregulation of E-cadherin and signs of aggressiveness or unresponsiveness to treatment in hormone-producing PitNETs [14, 22–25]. However, we have previously shown that this is not necessarily the case in gonadotroph NF-PitNETs, where the tumours presenting nuclear E-cadherin, associated with less membranous E-cadherin, were less likely to go through re-intervention than tumours not presenting nuclear E-cadherin [26]. The distribution of E- and N-cadherin in normal pituitary cells has only been investigated in a few studies, but the results point to a different distribution of these cadherins between the different pituitary cells [25, 27].

In this study, we aimed to characterise the distribution of E- and N-cadherin in corticotroph, PIT1 and null-cell NF-PitNETs, and link it to the tumour behaviour. We hypothesised that the E- and N-cadherin distribution in these subgroups would differ from the previously presented gonadotroph NF-PitNETs [26]. In addition, we compared the distribution of E- and N-cadherins between functioning and non-functioning corticotroph PitNETs [23].

## Methods

### Patients

Patients (*N* = 135) operated for clinically NF-PitNETs between 1998 and 2009, where tissue for tissue micro array (TMA) analyses was available, were selected from a larger retrospective cohort of patients (*N* = 194). Thirty of these tumours were classified as corticotroph, PIT1 or null-cell NF-PitNETs. All patients underwent the operation at the same tertiary referral centre. Pre-operative MRI was available for ten, four and three of the corticotroph, PIT1 and null-cell NF-PitNETs, respectively. The volume was calculated using the slice area method (Cavalieri’s principle), as previously described. Invasiveness was measured in accordance with the Knosp–Steiner classification, where a grade >2 on either side was defined as an invasive tumour [28]. All tumours were clinically classified as non-functioning at the time of surgery.

Patients with Cushing’s disease from a previously established TMA were used to compare cadherin expression between corticotroph functioning PitNETs and corticotroph NF-PitNETs. Patients with Nelson’s syndrome were included in the previously established cohort, while only adult patients with corticotroph functioning PitNETs were used for the present comparison. Moreover, only limited clinical data (gender, age, tumour size (micro/macro)) were available for the corticotroph functioning PitNETs [23, 29].

A new intervention less than 12 months after the primary surgery, re-operation or post-operative radiotherapy was considered as adjuvant to the primary surgery, and not included in the analyses of re-intervention. Re-intervention, more than 12 months after the primary surgery, included both radiation and surgery. One out of 30 patients died within 12 months after the primary pituitary surgery and was not included in the analyses concerning re-intervention.

### Histopathology and immunohistochemistry

The original haematoxylin eosin stained sections from all tumours were reviewed to confirm the presence of pituitary tumour tissue. TMAs were constructed containing replicate 1 mm cores from formalin-fixed paraffin embedded tissue samples from representative areas [29, 30].

Immunohistochemical classification was based on the expression of the anterior pituitary lobe hormones (FSH, LH, ACTH, GH, TSH, PRL and alpha subunit) and the transcription factors (SF1, TPIT and PIT1), as previously described [7, 31]. The tumours were classified into four groups based on their cell line of origin: Gonadotroph NF-PitNETs (SF1), corticotroph NF-PitNETs (TPIT), PIT1 NF-PitNETs and null-cell NF-PitNETs (not staining for anterior pituitary hormones or transcription factors). PIT1 positive group was heterogeneous, including four tumours expressing PRL, one expressing PRL and GH, two tumours expressing alpha-SU only and a peculiar case expressing TSH in a proportion of the cells and FSH, LH, and/or alpha-SU in scattered cells. In the last-mentioned case, PIT1 was strongly positive in the nuclei of all tumour cells, leading to the classification of the tumour as PIT1 positive. However, a weak SF1 staining was also observed. For purposes of statistical analyses, these uncommon cases were grouped together in the PIT1 positive group.

Three antibodies were used for the IHC analyses, targeting the cadherins: antibody towards the extra-cellular domain of E-cadherin (Abcam ab1416, RRID: AB_300946, mouse monoclonal, clone HECD-1), antibody towards the intra-cellular domain of E-cadherin (BD Transduction Laboratories, RRID: AB_397581, mouse monoclonal, clone 36/E-Cadherin), and antibody towards N-cadherin (Abcam ab98952, RRID: AB_10696943, mouse monoclonal, clone 5D5). The analyses were performed using the DAKO EnVision Flex+ system (K8012; DAKO, Glostrup, Denmark) and DAKO Autostainer. We have previously reported immunohistochemical analyses performed on the cohorts of functioning corticotroph tumours and non-functioning gonadotroph tumours [23, 26]. The IHC analyses with all three antibodies were performed on the extended NF-PitNET cohort, including silent corticotroph, silent PIT1 and null-cell PitNETs. In addition, immunohistochemistry with antibody towards N-cadherin was performed on nine of the 17 corticotroph functioning tumours that were available for analyses from the previous cohort of functioning corticotroph tumours. The same antibodies and the same staining platform were used for all the cohorts. However, the IHC analyses on the functioning corticotroph PitNETs and the NF-PitNETs were performed at different time intervals, in different diagnostic laboratories, using different instruments and batches of monoclonal antibodies. This required an optimisation of the IHC protocols in order to obtain the same intensity of the positive immunolabeling for comparison across the tumour cohorts. Thus, different dilutions of the antibody towards intra-cellular E-cadherin were used: 1:1000 in the cohort of functioning corticotroph tumours [23] and 1:300 in the current extended cohort and in the gonadotroph tumours [26]. The concentration of other antibodies was the same for all stainings. Positive controls routinely used in the respective IHC laboratories were also used in our studies, skin biopsy demonstrating distinct membrane staining in keratinocytes in the previous studies [23, 26], and a ductal breast carcinoma specimen in the analyses performed on the current tumour cohort. Negative controls were obtained by omitting the primary antibody.

The IHC stainings for E- and N-cadherin were scored using the immunoreactive score (IRS), as previously described [32, 33]. IRS is the product of the proportion of immunoreactive cells (0: 0%; 1: <10%; 2: 10–50%; 3: 51–80%; 4: >80%) and the staining intensity (0: no staining; 1: weak staining; 2: moderate staining; 3: strong staining) [32]. The IRS was stratified into negative (IRS 0–1), low (IRS 2 to 3), medium (IRS 4 to 8) and high (IRS 9 and 12) for some of the comparisons [34].

The same pathologist (O.C.-B.) performed all the immunoreactive scoring.

The results in the present study were compared to previously published results on E- and N-cadherin in gonadotroph NF-PitNETs [26] and E-cadherin in corticotroph functioning PitNETs [23]. The E-cadherin staining of the corticotroph functioning PitNETs was re-evaluated and quantified using the IRS system for the present study.

### Statistics

Statistical analyses for group comparison were performed with *χ*^2^ and Fischer’s exact test for nominal data, and with Mann–Whitney *U* and Kruskal–Wallis for all continuous data. Group differences were considered statistically significant at the 5% significance level. All statistical tests were two-sided tests.

Due to small numbers in the group of PIT1 and null-cell NF-PitNETs, statistical significance was mainly presented for the comparison of the corticotroph NF-PitNETs in relation to the gonadotroph NF-PitNETs.

## Results

Thirty NF-PitNETs were available for the present analyses; 18 were classified as corticotroph NF-PitNETs (TPIT positive), 8 as PIT1 NF-PitNETs, and 4 as null-cell NF-PitNETs (Table 1A). One hundred and five gonadotroph NF-PitNETs were used for comparison with the remaining subgroups of NF-PitNETs [26]. For one corticotroph NF-PitNET, the amount of the representative tumour tissue was unfortunately not sufficient for the analysis of the extra-cellular domain of E-cadherin.

**Table 1:** Comparison of subgroups of NF-PitNETs based on staining for anterior pituitary hormones and cell-linage specific transcription factors

	Corticotroph NF-PitNETs	PIT1 NF-PitNETs	Null-cell NF-PitNETs	Gonadotroph NF-PitNETs	All NF-PitNETs	*P* values
*A*						
Female (%)	9 (50)	5 (63)	1 (25)	33 (31)	48 (36)	0.13
Age	56 (50–70)	45 (26–58)*	68 (60–73)	60 (51–72)	59 (50–71)	0.30
Follow-up months	113 (98–176)	140 (117–168)	116 (100–145)	126 (99–158)	124 (99–159)	0.92
Re-intervention^a^	6 (35)	2 (25)	1 (25)	35 (35)	44 (34)	1.00
Adjuvant intervention	3 (18)	1 (13)	0 (0)	6 (6)	10 (7)	0.13
Tumour vol (mm^3^)^b^	6115 (2555–14,389)	2424 (1853–5963)	3440 (2104–7451)	6613 (4090–11,171)	6340 (3645–10,712)	0.48
Tumour invasion^b^	5 (50)	0 (0)	1 (25)	20 (31)	26 (43)	0.74
*B*						
Extrac. E-cadh IRS	0 (0–2)^c^	1.5 (0–3.5)**	0 (0–0)	0 (0–0)	0 (0–0)	0.001
Intrac. E-cadh IRS	9 (6–12)	7.5 (1.5–11.3)	9 (6.75–9)*	6 (4–6)	6 (4–9)	<0.001
Nuclear E-cadh	3 (17)	4 (50)	1 (25)	77 (73)	85 (63)	<0.001
N-cadh IRS	12 (11.25–12)	12 (8–12)^c^	12 (9.75–12)	12 (12–12)^c^	12 (10.5–12)	0.66
Total	18	8	4	105	135	

Results on gonadotroph NF-PitNETs have been presented previously and are used for comparison to the other subgroups [26]. *P* values are given for the comparison of corticotroph and gonadotroph NF-PitNETs. Median and quartiles are given for continuous data, while number and percentage of total are given for nominal data. Significant difference between PIT1 and null-cell NF-PitNETs and gonadotroph NF-PitNETs are marked with *p value < 0.05, **p value ≤ 0.001. **A** Re-intervention earlier than 12 months after primary surgery is defined as adjuvant treatment complementary to primary surgery. ^a^Six patients died within 12 months after surgery (one corticotroph and five gonadotroph NF-PitNETs). These are not included in the calculations concerning re-intervention. ^b^Pre-operative MRI was available for ten corticotroph, four PIT1 and three null-cell NF-PitNETs. **B** Comparison of the distribution of E-and N-cadherin in NF-PitNETs. ^c^Staining for the extra-cellular domain of E-cadherin was missing in one of the corticotroph NF-PitNETs. Staining for N-cadherin was missing in one PIT1 NF-PitNET

All thirty NF-PitNETs were macroadenomas. Indication for re-intervention was based on a clinical decision in all cases. Nine patients underwent either radiation or surgery more than 12 months after the primary surgery (Table 1A), and four of these (three corticotrophs and one PIT1 NF-PitNETs) had more than one re-intervention. Four patients (all with corticotroph NF-PitNETs) went through radiation, either as a secondary or tertiary intervention. Three patients (all with corticotroph NF-PitNETs) went through radiation within 12 months as an adjuvant treatment to primary surgery. One patient (PIT1 NF-PitNET) went through both surgery adjuvant to primary treatment and additional re-intervention more than 12 months after the primary surgery.

There were 17 tissue samples from corticotroph functioning PitNETs available for comparison with the corticotroph NF-PitNETs. Nuclear E-cadherin and intra- and extra-cellular E-cadherin have previously been presented for these tumours [23]. Twelve out of seventeen corticotroph functioning PitNETs were characterised as microadenomas (largest diameter less than 10 mm), while five were characterised as macroadenomas (largest diameter more than 10 mm).

### Distribution of E- and N-cadherin in subgroups of NF-PitNETs

The corticotroph, PIT1 and null-cell NF-PitNETs all showed a low IRS for the extra-cellular domain of E-cadherin and a medium to high staining for the intra-cellular domain of E-cadherin. All subgroups had a high IRS for N-cadherin (Table 1B).

**Fig. 1:**
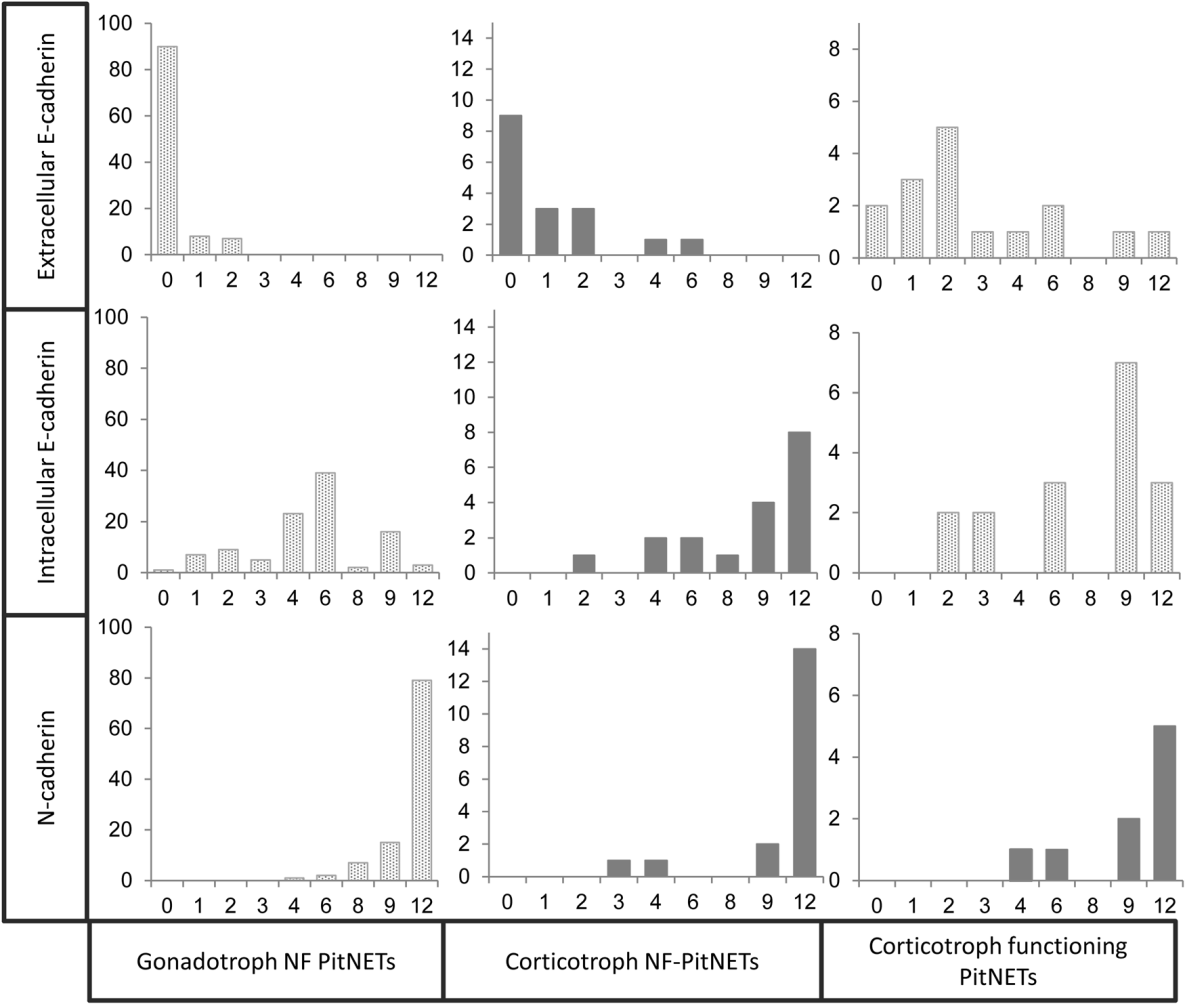
Comparison of immunoreactive score (IRS) for E- and N-cadherin in corticotroph NF-PitNETs. The IRS along the X-axis, and number of tumours along the Y-axis for corticotroph NF-PitNETs compared to gonadotroph NF-PitNETs and corticotroph functioning PitNETs, data on the two latter have for the most part been presented previously and are indicated with patterned columns [23, 26]. The corticotroph NF-PitNETs presented a higher IRS for the intra-cellular and extra-cellular domain of E-cadherin, compared to the gonadotroph NF-PitNETs (*p* < 0.001 and *p* = 0.001, respectively). They presented a lower IRS for the extra-cellular domain of E-cadherin compared to the corticotroph functioning PitNETs (*p* = 0.01). Staining for N-cadherin in corticotroph functioning tumours has not been performed previously and was only available in nine tumours

The corticotroph NF-PitNETs demonstrated a stronger staining for the extra-cellular domain of E-cadherin than the gonadotroph NF-PitNETs (median 0 (IQR 0–2) and median 0 (IQR 0–0), respectively, *p* = 0.001, Table 1B). However, the overall staining was weak, with 12 and 3 out of 17 available tumours showing a negative (IRS 0–1) or low IRS (2–3) (Fig. 1). The staining for the intra-cellular domain of E-cadherin was strong in the corticotroph NF-PitNETs, and significantly higher than in the gonadotroph NF-PitNETs (median 9 (IQR 6–12) and median 6 (IQR 4–6), respectively, *p* < 0.001, Table 1B) [26].

The PIT1 NF-PitNETs showed a higher IRS for the extra-cellular domain of E-cadherin (median 1.5 (IQR 0–3.5), *p* value < 0.001, Table 1B), but we found no statistical difference in IRS for the intra-cellular domain of E-cadherin and N-cadherin compared to the gonadotroph NF-PitNETs (Table 1B).

### Nuclear E-cadherin in subgroups of NF-PitNETs

Eight tumours presented nuclear staining for the intra-cellular domain of E-cadherin (Table 1B). A lower proportion of corticotroph NF-PitNETs presented nuclear E-cadherin compared to the gonadotroph NF-PitNETs (3 out of 18 vs 77 out of 105, respectively, *p* < 0.001, Table 1B) [26].

**Fig. 2:**
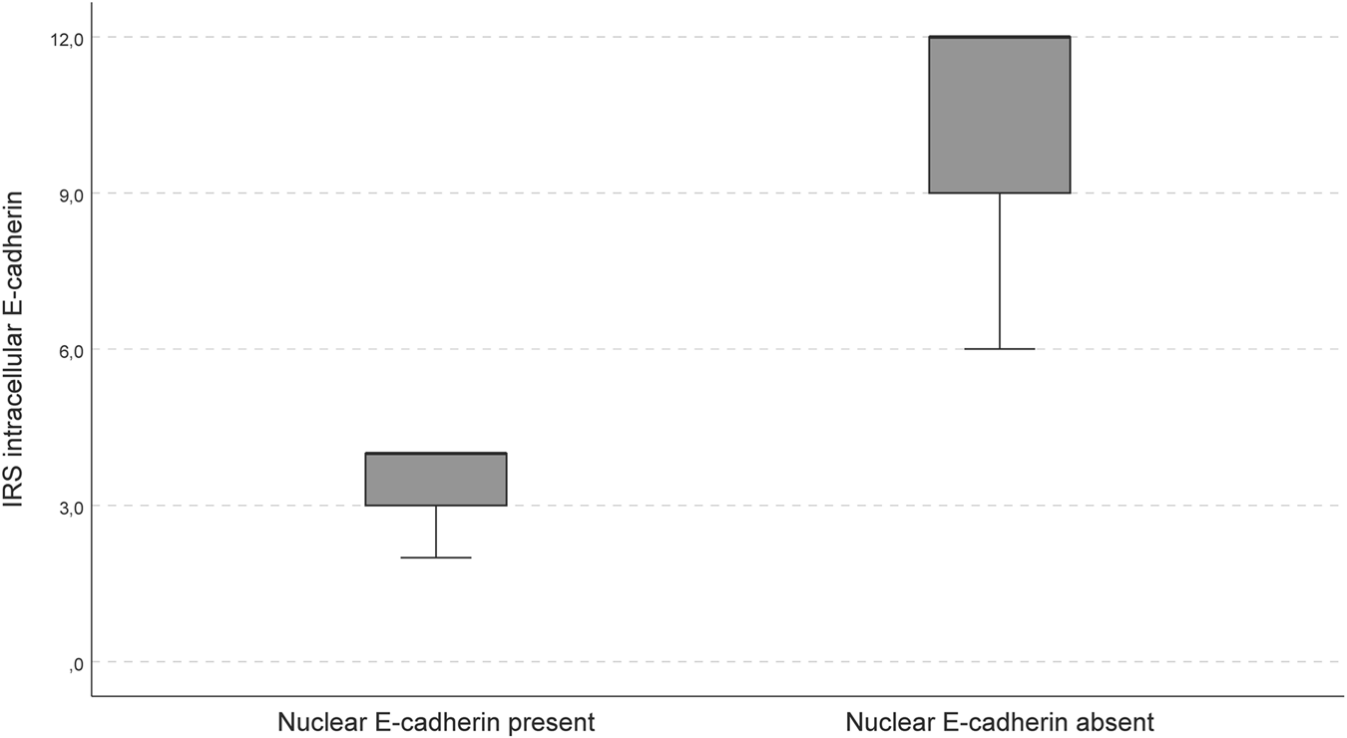
Association between nuclear E-cadherin and membranous E-cadherin in corticotroph NF-PitNETs. Three out of 18 corticotroph NF-PitNETs presented nuclear E-cadherin. The membranous staining for the intra-cellular domain of E-cadherin was significantly different (*p* **=** 0.005) between the tumours presenting nuclear E-cadherin and those not. The three corticotroph NF-PitNETs with nuclear presentation showed an IRS of 2, 4 and 4 for the intra-cellular domain of E-cadherin, and the 15 corticotroph NF-PitNETs without nuclear E-cadherin showed a median IRS of 12 (IQR 9–12)

**Fig. 3:**
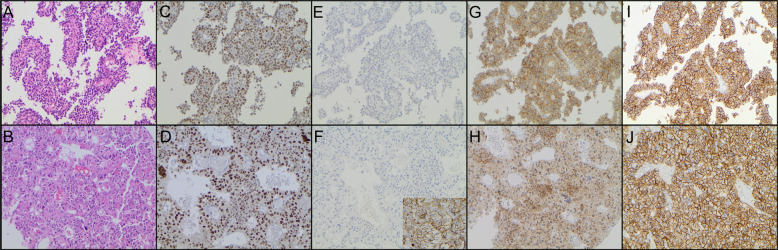
Morphological and immunohistochemical features of two corticotroph NF-PitNETs with differential expression of the intra-cellular domain of E-cadherin. Each row is representing one tumour. **A**, **B** Haematoxylin eosin staining. **C**, **D** Positive immunolabeling for the transcription factor TPIT. **E**, **F** Lack of immunolabeling for extra-cellular domain of E-cadherin in the tumours (positive control from a ductal breast carcinoma is shown in the insert). **G** Strong membranous expression of the intra-cellular domain of E-cadherin (IRS 12) without nuclear protein expression. **H** Moderate expression of the intra-cellular domain of E-cadherin in a minor proportion of the tumour cells (IRS 2) associated with a weak to moderate nuclear protein expression. **I**, **J** Strong cell membrane expression of N-cadherin in both tumours (IRS 12)

There was an association between the presence of nuclear E-cadherin and the staining score for the intra-cellular domain of E-cadherin located at the cell membrane (median 4 (IQR 0.5–6) and 9 (9–12), for tumours with and without nuclear E-cadherin, respectively, *p* < 0.001). This association was also present when analysing the corticotroph NF-PitNETs separately (IRS 2, 2 and 4 and median 9 (IQR 9–12), for tumours with and without nuclear E-cadherin, respectively, *p* = 0.005, Figs. 2 and 3).

All but one tumour (IRS 2) with nuclear E-cadherin presented no staining (IRS 0) for the extra-cellular domain of E-cadherin, while 8 of 22 tumours without nuclear E-cadherin showed an IRS > 2.

### Cadherin and aggressiveness in subgroups of NF-PitNETs

None of the eight tumours that presented nuclear E-cadherin went through re-intervention or adjuvant intervention, while nine (43%) and four (17%) out of the adenomas not presenting nuclear E-cadherin (*N* = 21) were in need of re-intervention or adjuvant intervention, respectively. There was a weak significant difference in re-intervention between the two groups (*p* = 0.03). One person with less than 12 months of follow-up was excluded from this analysis. The tumour from this patient did not present nuclear E-cadherin.

### Comparison of functioning and non-functioning corticotroph PitNETs

There was no statistical difference in gender and age distribution between corticotroph NF-PitNETs (data presented in Table 1A) and corticotroph functioning PitNETs (12 females, median age 49 (IQR 37–60), *p* = 0.31 and *p* = 0.11, respectively).

The IRS of intra-cellular E-cadherin (median 9, IQR 4.5–9), N-cadherin (median 12, IQR 7.5–12) and the proportion of tumours with nuclear E-cadherin (29%) in the corticotroph functioning PitNETs did not appear statistically different (*p* = 0.20, *p* = 0.40 and *p* = 0.44, respectively) from the corticotroph NF-PitNETs (data presented in Table 1B). The IRS for the extra-cellular domain of E-cadherin differed between the functioning and non-functioning corticotroph PitNETs (median 2 (IQR 1–5.5) and 0 (0–2), respectively, *p* = 0.01). There were two tumours showing a high IRS (9 and 12) for the extra-cellular domain of E-cadherin in the functioning group, while the remaining 15 presented an IRS of six or less. However, the difference between the functioning and non-functioning corticotroph PitNETs remained significant (*p* = 0.03) when excluding these two tumours.

## Discussion

In this study, we found that the IRS for the extra-cellular domain of E-cadherin was low, the intra-cellular domain of E-cadherin was moderate to high, and N-cadherin was overall high in the corticotroph, PIT1 and null-cell NF-PitNETs. Moreover, the corticotroph NF-PitNETs demonstrated a higher expression of intra-cellular and extra-cellular E-cadherin, and fewer tumours with nuclear E-cadherin compared to the gonadotroph NF-PitNETs. NF-PitNETs with nuclear E-cadherin presented lower IRS for the intra-cellular domain of E-cadherin than tumours without nuclear E-cadherin, which is similar to the pattern previously presented in the gonadotroph NF-PitNETs [26]. We did not find a statistical difference in the presence of nuclear E-cadherin, intra-cellular E-cadherin and N-cadherin, when comparing functioning and non-functioning corticotroph tumours.

The staining for N-cadherin was strong for the entire cohort, without subgroup difference. N-cadherin is prevalent in non-epithelial tissue. In epithelial tumours, N-cadherin has been associated with a transition towards a more mesenchymal tumour phenotype, with more invasive and motile features [18]. However, N-cadherin has been found in normal pituitary tissue and reduced expression associated with invasiveness for some pituitary tumours [27, 35]. The relation between N-cadherin and tumour aggressiveness in other neuroendocrine tumours has been variable [36, 37]. As an example, N-cadherin was found to function as a tumour suppressor in pancreatic cancer in mouse models [38]. Considering that PitNETs are benign tumours with slow growth, we hypothesise that the classical role for N-cadherin, where high levels are linked to a more aggressive phenotype, is not plausible in NF-PitNETs.

Overall, the IRS for the extra-cellular domain of E-cadherin was low in the subgroups of NF-PitNETs. Loss of E-cadherin as a membranous adherence protein has been associated with a more aggressive phenotype in functioning PitNETs [22, 23, 25, 39, 40]. However, both high and low E-cadherin expressions have been found in normal epithelial tissue [25, 41]. Studies have suggested that both up- and downregulation of E-cadherin from the normal cell state might be associated with a more aggressive clinical course, and that the process of EMT is not dependent on a reduction in E-cadherin [41, 42]. Information on the distribution of cadherins in normal pituitary cells is scarce, due to the lack of normal pituitary tissue available for investigation. However, a few studies have demonstrated that both E- and N-cadherin are present in the normal anterior pituitary [25, 27, 35]. Results from a single study that presented E- and N-cadherin from three normal pituitary samples with simultaneous staining for anterior pituitary hormones showed that gonadotroph, corticotroph and thyrotroph cells presented a low expression of E-cadherin and high expression of N-cadherin in contrast to somatotroph and lactotroph cells. This study used a monoclonal antibody directed against the extra-cellular domain of E-cadherin [25].

For the corticotroph NF-PitNETs, we found a higher expression for the extra-cellular domain of E-cadherin compared to the gonadotroph NF-PitNETs, and a more similar pattern of N-cadherin, intra-cellular and nuclear E-cadherin compared to the corticotroph functioning PitNETs. Nishioka et al. compared NF-PitNETs with functioning PitNETs of the same cell-origin and found that there were similar tumour features within the same cell-lineages, independent of functionality [5]. The previous study on the corticotroph functioning tumours found that corticotroph functioning microadenomas had stronger E-cadherin staining than corticotroph functioning macroadenomas [23]. There were only macroadenomas in the corticotroph non-functioning cohort, potentially influencing the result. Moreover, the previous study included patients with Nelson’s syndrome [23]; these patients were not included in the present comparison.

The IRS of the intra-cellular domain of E-cadherin located at the membrane was significantly lower in tumours with nuclear E-cadherin than in tumours without, in NF-PitNETs. Similar findings have previously been shown in gonadotroph NF-PitNETs [26], and also in functioning PitNETs [23, 43]. This pattern might be caused by a translocation of the cytoplasmic domain of E-cadherin from the cell membrane to the nucleus [44]. However, the mechanism has not been tested in pituitary cell lines. The presence of nuclear E-cadherin has been associated with a loss of membranous E-cadherin and hence, a more aggressive tumour behaviour [45]. However, the presence of nuclear E-cadherin has been found in normal non-pituitary tissue, and associated with less aggressive phenotypes in some epithelial cancers [46, 47]. The turnover of membranous E-cadherin is faster than the transcriptional process of E-cadherin; hence, there is a continuous recycling and degradation of E-cadherin in cells [48]. Previous studies have pointed out different endocytic pathways for membranous E-cadherin [48]. The possible mechanism directing the intra-cellular portion of the E-cadherin to the nucleus in the pituitary cells is so far unclear.

None of the tumours presenting nuclear E-cadherin went through re-intervention, whereas nine of the remaining tumours did. The role of nuclear E-cadherin is not fully elucidated, though studies have shown that it might constitute regulatory mechanisms in gene transcription and potentially regulate apoptotic processes [44, 49]. Furthermore, it is not known whether these findings are alienable to pituitary cells; hence, cell studies are needed to explore these mechanisms.

### Limitations

The study was of retrospective character, and IHC analyses were performed where tissue samples were available, and thus potentially subjected to inclusion bias. The PIT1 and null-cell NF-PitNETs are rare; therefore, generating power for statistical comparison with the larger groups was difficult. The results for these groups should be interpreted cautiously. In addition, the PIT1 group was a heterogeneous group, containing tumours with different hormone characteristics. The number of corticotroph NF-PitNETs with nuclear staining for E-cadherin was low, which in itself is an interesting finding. However, it weakens the statistical comparison of nuclear presence and aggressiveness within the corticotroph NF-PitNETs. NF-PitNETs are operated due to mass effect on the surrounding tissue, while tumours with less growth potential are not available for tissue analyses. This might reduce the range in aggressiveness of tumours investigated and give a homogeneous group. Only seven tissue samples were available for RT-qPCR analyses in the corticotroph NF-PitNET group; consequently, data were not presented. Lastly, EMT is a dynamic and complex process and cannot be clearly described with a few molecular markers alone; hence, dynamic studies with characterisation of cell features would be of value to characterise the EMT process in pituitary cells [21].

## Conclusion

The investigated non-functioning PitNETs presented a high IRS for N-cadherin, low IRS for the extra-cellular domain of E-cadherin, and a variable IRS for the intra-cellular domain of E-cadherin. Considering their mostly benign clinical course, the downregulation of E-cadherin does not seem to be a driver of aggressiveness in these tumours. Nuclear E-cadherin was associated with a lower IRS of the intra-cellular domain of E-cadherin than tumours without nuclear E-cadherin. We hypothesise that E-cadherin is translocated from the membrane to the nucleus in NF-PitNETs, where it might influence the biological behaviour of the tumours. This, however, needs to be validated in pituitary cell studies.
